# Rapid and Highly Efficient Separation of i-Motif DNA Species by CE-UV and Multivariate Curve Resolution

**DOI:** 10.1021/acs.analchem.3c01730

**Published:** 2023-10-02

**Authors:** Laila Bchara, Ramon Eritja, Raimundo Gargallo, Fernando Benavente

**Affiliations:** † Department of Chemical Engineering and Analytical Chemistry, University of Barcelona, Marti i Franquès 1-11, E-08028 Barcelona, Spain; ‡ Institute for Advanced Chemistry of Catalonia (IQAC−CSIC), CIBER-BBN, Jordi Girona 18-26, E-08034 Barcelona, Spain; § Institute for Research on Nutrition and Food Safety (INSA·UB), University of Barcelona, Av. Prat de la Riba 171, E-08921 Santa Coloma de Gramenet, Spain

## Abstract

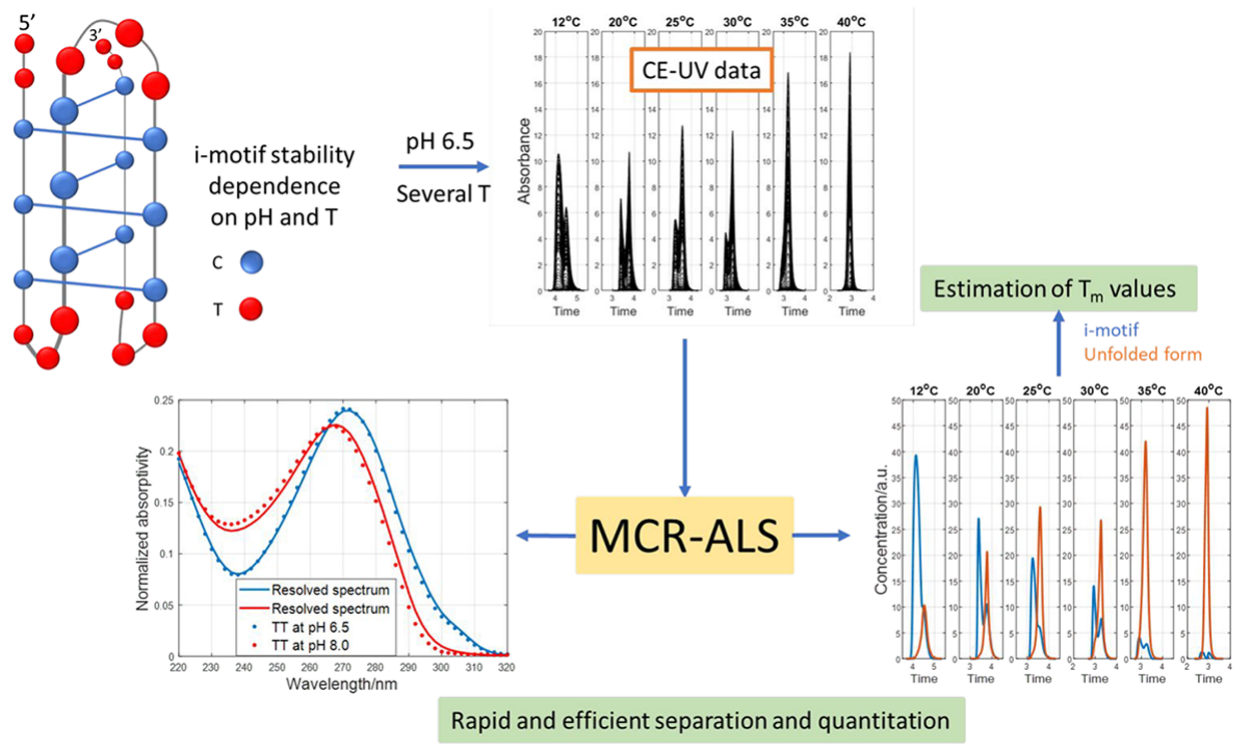

The i-motif is a class of nonstandard DNA structure with potential biological implications. A novel capillary electrophoresis with an ultraviolet absorption spectrophotometric detection (CE-UV) method has been developed for the rapid analysis of the i-motif folding equilibrium as a function of pH and temperature. The electrophoretic analyses are performed in reverse polarity of the separation voltage with 32 cm long fused silica capillaries permanently coated with hydroxypropyl cellulose (HPC), after an appropriate conditioning procedure was used to achieve good repeatability. However, the electrophoretic separation between the folded and unfolded conformers of the studied cytosine-rich i-motif sequences (i.e., TT, Py39WT, and nmy01) is compromised, especially for Py39WT and nmy01, which result in completely overlapped peaks. Therefore, deconvolution with multivariate curve resolution-alternating least-squares (MCR-ALS) has been required for the efficient separation of the folded and unfolded species found at different concentration levels at pH 6.5 and between 12 and 40 °C, taking advantage of the small dissimilarities in the electrophoretic mobilities and UV spectra levels. MCR-ALS has also provided quantitative information that has been used to estimate melting temperatures (*T*_m_), which are similar to those determined by UV and circular dichroism (CD) spectroscopies. The obtained results demonstrate that CE-UV assisted by MCR-ALS may become a very useful tool to get novel insight into the folding of i-motifs and other complex DNA structures.

DNA may form complex structures apart from the well-known duplex structure first proposed by Watson and Crick. Among them, the intercalated motif (i-motif) structure is formed by cytosine-rich sequences, which may be found at the end of telomeres, centromeres, and near the promoter regions of several oncogenes.^1−3^ Structurally, the i-motif consists of two parallel duplexes intercalated in a parallel manner (Figure 1). These duplexes are held together through hydrogen bonds between pairs of cytosine bases. The protonation of one of the bases at N3 (the p*K*_a_ of which is around 4.5) produces the formation of a cytosine·cytosine^+^ (C·C^+^) base pair that is stabilized by three hydrogen bonds (Figure 1a), resulting in folded structures (Figure 1b–d).^4−7^ Consequently, the stability of this folded structures depends largely on the number of C·C^+^ base pairs, as well as on the nature and length of the loops.^8,9^ Furthermore, stability is greatly enhanced at pH values near the p*K*_a_ of cytosine, while it decreases at neutral pH values. Other external factors influencing the i-motif stability are the ionic strength and the temperature. The i-motif structure has become a subject of great interest because of its potential role *in vivo*,^10−12^ as well as for their application in other fields, such as the development of nanomotors^13^ or analytical sensors,^14^ among others. Many of these applications make use of pH-dependent folding of this structure. Hence, as an example, the folding/unfolding equilibria of i-motif species has been applied to create nanopores, the size of which is depending on pH.^15^ Also, labeling the i-motif forming sequences with appropriate fluorescent and quencher moieties may be used as pH-sensors near the biological pH in cell and *in vivo* media.^16,17^

**Figure 1:**
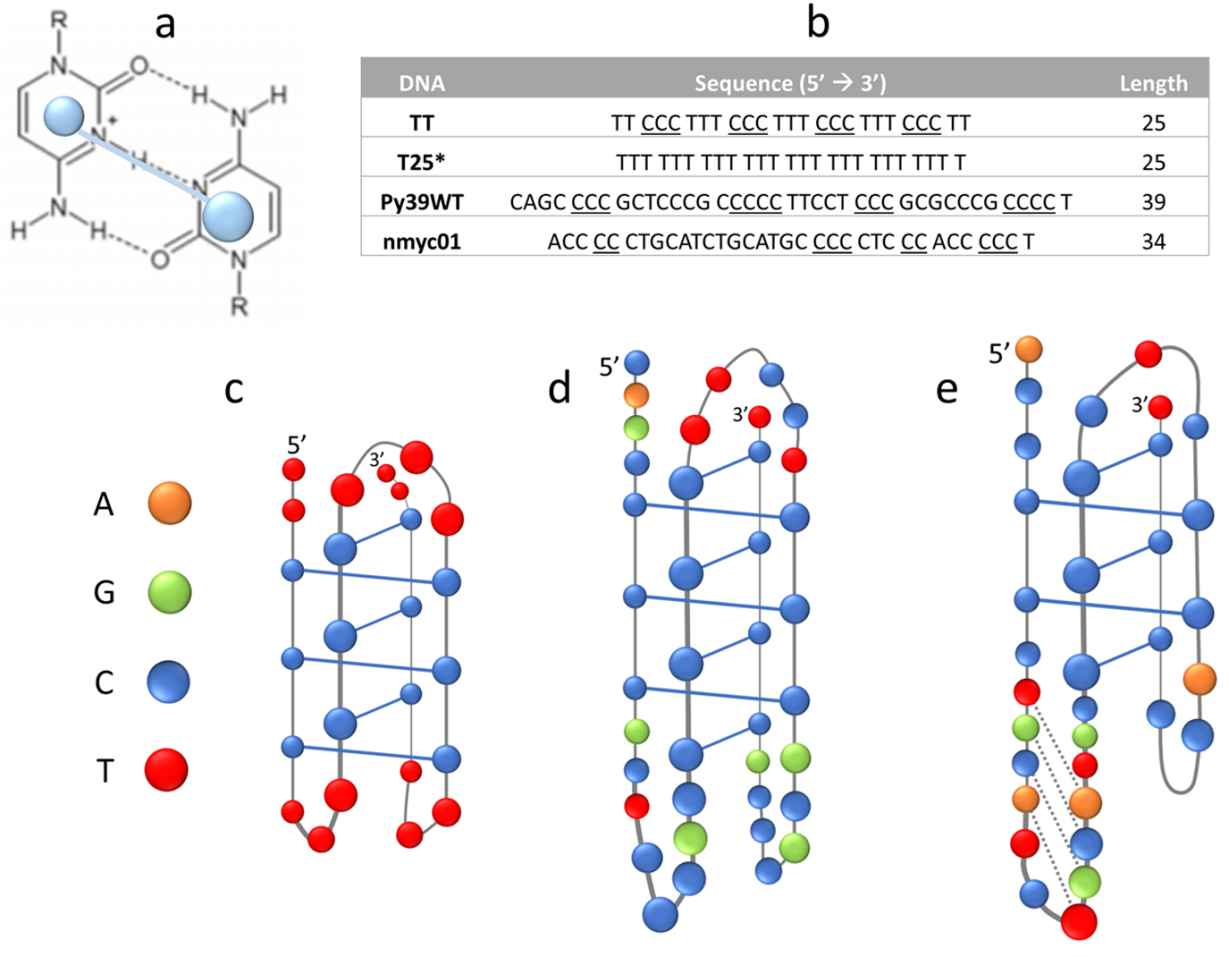
(a) C·C^+^ base pair stabilized by three hydrogen bonds between a deprotonated and a protonated cytosine base. (b) Studied DNA sequences. The cytosine-rich tracts involved in the core of the i-motif schemes have been underlined and T25* sequence was used as unfolded control. (c) Hypothetical folding of the i-motif formed by TT.^4^ (d) Experimentally determined folding of the i-motif formed by Py39WT.^5^ (e) Hypothetical folding of the i-motif formed by nmyc01.^6^

The study of the conformational equilibria involving i-motif structures is usually carried out by means of spectroscopic and separation techniques. Circular dichroism (CD) and ultraviolet (UV) absorption spectroscopies are used to monitor unfolding induced by temperature changes.^18^ Whereas these techniques provide a whole view of the unfolding process, nuclear magnetic resonance (NMR) allows a local view of the changes accompanying these conformational processes.^19^ Complementary information may be obtained from separation techniques such as size exclusion chromatography (SEC) or polyacrylamide gel electrophoresis (PAGE). SEC has been shown to be useful to detect the folding of cytosine-rich sequences into i-motifs, but low separation capacity and resolution have been achieved.^20^ PAGE is a well established technique that provides appropriate separations and band profiles from which information about the species in equilibrium or the molecular mass can be estimated. However, it is a labor-intensive and time-consuming technique with a limited separation capacity. As a result, resolution, reproducibility, throughput, and performance in quantitative analysis are very limited. Capillary electrophoresis (CE) may overcome some of the difficulties found by SEC or PAGE. CE is a high resolution microseparation technique with well-known advantages from the point of view of low reagent, solvent, and sample consumption, fast speed of analysis, fully automated instrumentation, and easiness of operation.^21^

In the most basic CE mode, namely, capillary zone electrophoresis (CZE), charged analytes are separated according to their electrophoretic mobilities in a capillary filled with a background electrolyte (BGE) of a certain pH value. Electrophoretic mobilities are proportional to the analyte charge-to-hydrodynamic radius ratios, which depend on their acid–base dissociation constants in case of ionizable compounds, and molecular mass values.^22^ CZE has been recently proposed to identify the folding of cytosine-rich sequences into i-motifs, based on the slight differences on migration found between the folded and unfolded species.^23^ However, the proposed method has not been applied to investigate the conformational equilibria of i-motifs near the pH-transition midpoints (pH_1/2_) between the folded and the unfolded species or the effect of temperature on these conformational equilibria. In addition, other important issues, such as the repeatability of the analyses or the limited separation resolution with complex i-motif structures, have been overlooked, blurring and underestimating the true potential of CZE for the rapid and efficient separation and characterization of i-motif species.

In this work, folding of three cytosine-rich sequences (TT, Py39WT, and nmyc01, Figure 1b–e) of different structural complexity, which have previously been shown to fold into i-motif structures under different pH and temperature conditions,^4−6^ are studied by CZE (hereinafter denoted as CE). The TT sequence contains 12 cytosine and 13 thymine bases. Because of the even number of cytosine bases, TT may fold into an i-motif stabilized by six C·C^+^ base pairs.^4^ The study with NMR spectroscopy of this structure has revealed the presence of additional T·T base pairs, which enhance the stability of the structure in the presence of pH or temperature changes. The Py39WT sequence is a thirty-nine-nucleotide long sequence that presents cytosine stretches of different length, as well as a variety of bases. It has been shown to form an i-motif structure stabilized by seven C·C^+^ base pairs,^5^ as well as a hairpin structure containing five Watson–Crick base pairs at neutral pH.^24,25^ Finally, the nmyc01 sequence presents four tracts of cytosine bases of disparate lengths and additional cytosine bases in the loops. It has been proposed to fold into an ensemble of i-motif structures stabilized by a maximum of five C·C^+^ base pairs. The nature of the bases in the loops allows the formation of additional base pairs. Therefore, some of the bases located in one of the loops of the i-motif may form a short hairpin stabilized by Watson–Crick hydrogen bonds.^6^

The CE-UV separation of the folded and unfolded i-motif species formed by TT, Py39WT, and nmyc01 is investigated as a function of pH and temperature. Short capillaries permanently coated with hydroxypropyl cellulose (HPC) combined with an appropriate conditioning procedure are proposed to achieve rapid and repeatable electrophoretic analyses in reverse polarity of the separation voltage with UV absorption spectrophotometric detection. However, the achieved electrophoretic separation between i-motif species is compromised, especially for the most complex sequences that result in highly overlapped peaks. In such a scenario, the application of multivariate curve resolution-alternating least-squares (MCR-ALS)^26−28^ is critical for the deconvolution and efficient separation of the i-motif species, taking advantage of the small differences in the electrophoretic mobilities and UV spectra levels. The obtained results demonstrate that CE-UV assisted by MCR-ALS may become a very useful tool to provide novel insight into the folding of i-motifs and other complex DNA structures.

## Experimental Section

### Reagents

The DNA sequences (Figure 1b) were obtained from Sigma-Aldrich (St. Louis, Missouri, U.S.A.). DNA strand concentration was determined by absorbance measurements (260 nm) at 90 °C using the molar absorption coefficients calculated using the nearest-neighbor method as implemented on the OligoCalc webpage.^29^ Before the experiments, DNA solutions were heated to 90 °C for 10 min and then allowed to reach room temperature and anneal overnight. All the chemicals for the preparation of BGEs and solutions were analytical reagent grade. K_2_HPO_4_, and NH_4_OH were purchased from Panreac AppliChem (Barcelona, Spain). HPC (100000 average molecular mass), HCl, KOH, and methanol were supplied by Merck (Darmstadt, Germany). Water was obtained using a Milli-Q purification system (Millipore, Molsheim, France).

### Background Electrolytes

BGEs were buffer solutions prepared from a 15 mM K_2_HPO_4_ solution adjusted to pH 6.0, 6.5, 7.0, and 7.5 with HCl. A phosphate buffer was chosen for three reasons. First, the second p*K*_a_ of phosphoric acid is around 7.1 at physiological conditions of temperature and ionic strength.^30^ Therefore, it is expected to present a relatively good buffer capacity between approximately pH 6.1 and 8.1. Second, its temperature coefficient is −0.0028 pH units·°C^1–^, which may result in a small pH variation with temperature in the range 12–40 °C. Finally, phosphate buffers have been widely used in CE-UV separations of a broad range of compounds and in spectroscopically monitored melting experiments of DNAs because of its low absorbance at short wavelengths.^18,21^ The use of phosphate buffers in electrophoretic separation and melting experiments allows a straightforward and reliable comparison of the results with both techniques.

### Apparatus and Instruments

All CE-UV experiments were performed on a 7100 CE instrument (Agilent Technologies, Waldbronn, Germany) with UV absorption diode array detection (DAD). The autosampler of the CE instrument was kept at the same temperature as the CE cartridge cassette using an external water bath (Minichiller 300, Peter Huber Kältemaschinenbau AG, Offenburg, Germany). Data acquisition was performed with ChemStation C.01.06 software (Agilent Technologies).

CD spectra were recorded on a Jasco J-810 spectropolarimeter equipped with a Julabo F-25/HD temperature control unit. UV spectra were recorded on an Agilent 8453 spectrophotometer. Hellma quartz covered cells (10 mm path length, 3000 mL volume) were used in both cases.

pH measurements were made with a Crison 2002 potentiometer and a Crison electrode 52–03 (Crison Instruments, Barcelona, Spain).

### Procedures

The 75 μm internal diameter (i.d.) × 365 μm outer diameter (o.d.) bare fused silica capillaries were provided by Polymicro Technologies (Phoenix, AZ, U.S.A.). CE-UV experiments were performed with capillaries internally coated with hydrophilic polymer HPC to avoid DNA adsorption on the bare fused silica inner walls. HPC permanent coating was prepared by stabilization of the coating layer at high temperature as described in a recent publication.^31^ Under optimized conditions, 32 cm total length (*L*_T_) capillaries were used for the CE-UV experiments. The UV detection window was made at 8.5 cm from the outlet (23.5 cm effective length, *L*_D_). Coating of the HPC capillaries, conditioning before the CE-UV analyses, and other details are described in Section S1.

For the melting experiments, the DNA solution in BGE, previously annealed overnight, was transferred to the cell and allowed to equilibrate at the initial temperature for 15 min. For UV-monitored melting experiments, spectra were measured at 2 °C intervals with a hold time of 3 min at each temperature, which yielded an average heating rate of approximately 0.6 °C·min^–1^. For CD-monitored melting experiments, ellipticity was measured at 285 nm, whereas spectra (210–310 nm) were measured every 5 °C, being the heating rate 0.5 °C min^–1^.

### Data Analysis

Single-wavelength raw electropherograms at 254 nm were processed for Gaussian fitting as described in Section S2. Additionaly, multivariate data analysis based on MCR-ALS was applied to multiwavelength raw electropherograms to resolve peaks from i-motif species not completely separated by CE.^26,32−34^ This methodology has also been applied to other aspects related with the investigation of chemical and conformational equilibria of nucleic acids using spectroscopic techniques,^35,36^ like melting experiments.^37^ A detailed description of the MCR-ALS data analysis is given in Section S2. ALS optimization was performed under non-negativity constraints for concentration and spectral profiles and spectral normalization (equal length).^28,34^ Before analysis of the experimental CE-UV data with MCR-ALS, the conditions applied for MCR-ALS were appropriately validated (Section S3).

## Results and Discussion

A CE-UV method recently described to analyze i-motif structures based on the slight differences on migration observed between the folded and unfolded species at 254 nm was our starting point for the analysis of the TT sequence.^23^ TT was analyzed first because it was simpler than Py39WT and nmyc01, which in addition to the i-motif structures could form other intramolecular structures stabilized by Watson–Crick base pairs of low stability. A different set of DNA sequences was analyzed in that original CE-UV method, using a 50 μm i.d. × 50 cm L_T_ bare fused silica capillary in normal polarity (cathode in the outlet) and a BGE of 25 mM HEPES, 5 mM KH_2_PO_4_, and 1 mM MgCl_2_, adjusted to pH 6.5 or 8.0. However, in our study, no DNA peaks were detected under these conditions, or they were randomly detected with different migration times (*t*_m_), peak areas, and shapes. These poor results were probably due to DNA adsorption on the inner wall of the bare fused silica capillary. Indeed, the high affinity of nucleic acids toward silica or related materials (e.g., glass diatomaceous earth, etc.) is well-known.^38^ Retention on the ionized silanol groups may be based on hydrogen bonds formation but mainly on electrostatic interactions through cation bridges, where cations (e.g., sodium ions) act as a bridge between the negatively charged phosphate DNA backbone and the negatively charged silanol groups on the inner capillary wall. As an alternative to bare fused capillaries, we explored capillaries coated with the hydrophilic polymer HPC, which have been widely described to prevent adsorption of proteins, DNA fragments, and several aromatic compounds.^21,31,39,40^ In HPC capillaries, the electroosmotic flow is suppressed, and the separation polarity must be reversed to detect the negatively charged DNA species. In addition, short HPC capillaries were used to minimize the total separation time with reduced washing steps (2 min) for capillary conditioning. Preliminary experiments using BGEs of 15 mM K_2_HPO_4_ at different pH and temperature values were promising, but still repeatability was not as expected, especially at those conditions where folded i-motif species were detected. This issue was definitively solved by extending the duration of the capillary conditioning steps to 10 min (Section S1). Under these conditions, repeatability of migration times and peak areas was appropriate for an accurate and reliable folding analysis by CE-UV (e.g., 0.4% and 0.5% of relative standard deviation for migration times and peak areas, respectively, analyzing a 15 μM TT sample with a BGE of 15 mM K_2_HPO_4_ at pH 6.5 and 12 °C, *n* = 3).

### Analysis of TT Sequence

**Figure 2:**
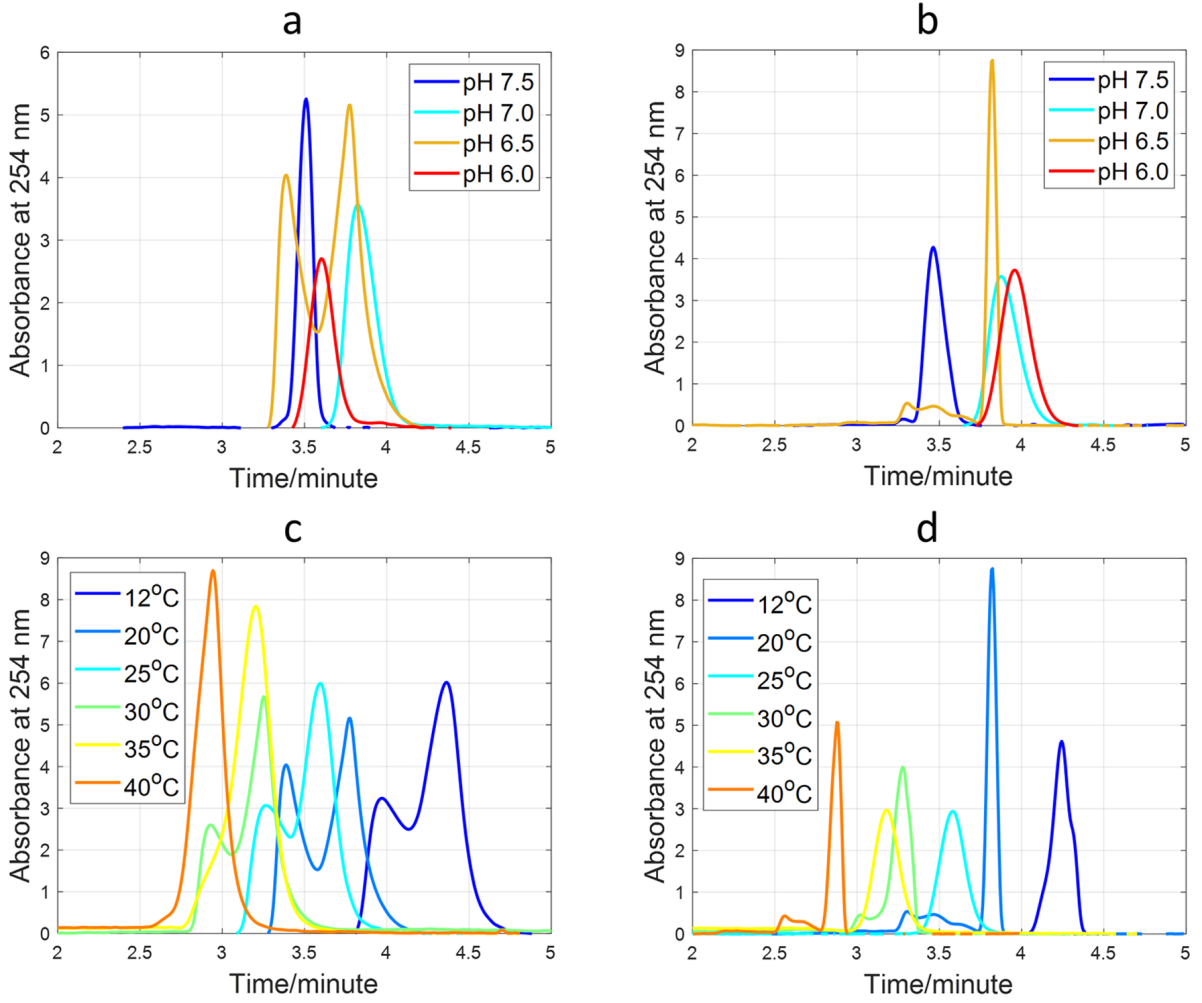
Electropherograms of 15 μM TT (a) and T25 (b) samples with a BGE of 15 mM K_2_HPO_4_ at pH values: 6.0, 6.5, 7.0, and 7.5 and 20 °C. Electropherograms of 15 μM TT (c) and T25 (d) samples with a BGE of 15 mM K_2_HPO_4_ at pH 6.5 and 12, 20, 25, 30, 35, and 40 °C.

The study was started investigating the TT sequence at a fixed *T* and different pH values, as pH is well-known to affect i-motif folding. Figure 2a shows the electropherograms of a 15 μM TT sample analyzed at pH values ranging from 6.0 to 7.5 and 20 °C. At pH 7.5 and 7.0, only one peak was observed. At both pH values, the *t*_m_ of the TT peaks agreed with those measured for T25 (Figure 2b and Table 1), which was a DNA sequence of the same length used as the unfolded control. Therefore, this fact suggested that TT was unfolded at pH 7.0 and 7.5. A different behavior was observed at pH 6.5. At this pH value, the electropherogram of TT showed two clear peaks, being the *t*_m_ of the second peak like that of the unfolded T25 (Figure 2b and Table 1). The faster migration to the anode of the folded i-motif species agreed with a more compact structure presenting a smaller hydrodynamic radius as observed by SEC,^20^ hence, with a greater electrophoretic mobility, if assumed that the overall negative charge was close for the folded and unfolded species. At pH 6.0, only one peak appeared in the electropherogram of TT corresponding to the folded species. For this reason, *t*_m_ was clearly smaller than that of T25 at the same pH value, which remained unfolded (Figure 2b and Table 1). Regarding the influence of pH on the electrophoretic mobility of the studied DNA sequences, the phosphodiester backbone (p*K*_a_ ∼ 1) and cytosine (p*K*_a_ ∼ 4.5) nucleobases were deprotonated in the studied pH range, while thymine (p*K*_a_ ∼ 9) deprotonation seemed to increase the negative global charge of the DNA sequences only from pH 7.5, resulting in greater electrophoretic mobilities and shorter *t*_m_ (see, for example, Figure 2b).

**Table 1:** Migration Times (*t*_m_) of TT and T25 at the Studied pH Values and Temperatures (*t*_m_ ± Standard Deviation, *n* = 3)^a^

BGE pH	7.5	7.0	6.5						6.0
temperature (°C)	20	20	40	35	30	25	20	12	20
T25 (unfolded)	3.46 ± 0.02	3.88 ± 0.03	2.90 ± 0.03	3.18 ± 0.04	3.27 ± 0.02	3.59 ± 0.04	3.77 ± 0.04	4.27 ± 0.04	3.96 ± 0.03
TT 1st peak (folded)	n.d.	n.d.	n.d.	2.87 ± 0.01	2.97 ± 0.02	3.28 ± 0.02	3.41 ± 0.03	4.01 ± 0.02	3.61 ± 0.02
TT 2nd peak (unfolded)	3.51 ± 0.03	3.82 ± 0.02	2.92 ± 0.02	3.19 ± 0.02	3.26 ± 0.02	3.61 ± 0.01	3.76 ± 0.03	4.35 ± 0.02	n.d.

a n.d.: not detected.

If the peaks appearing in the electropherogram of TT at pH 6.5 corresponded to the folded and unfolded forms of the TT sequence, it would be expected that the ratio between both peaks would change with temperature. In other words, it would be possible to use CE-UV as an approximation to the spectroscopically monitored melting experiments used to determine the thermal stability of folded DNA structures, such as the i-motif.^18^ Therefore, the TT sequence was analyzed at pH 6.5 and several temperatures (Figure 2c and Table 1). As expected at this pH value, the electropherograms of TT (Figure 2c) showed two peaks at 12, 20, 25, and 30 °C corresponding to the folded and unfolded species, and *t*_m_ were decreasing as a function of *T* due to the decrease in the BGE viscosity.^21^ At 35 °C, a shoulder was observed at around 2.87 min in addition to the main peak at 3.19 min. This shoulder disappeared at 40 °C, and only the peak corresponding to the unfolded species was observed. In contrast to TT, the electropherograms of T25 (Figure 2d and Table 1), showed one single peak at all temperatures in agreement with the detection of the unfolded species.

**Figure 3:**
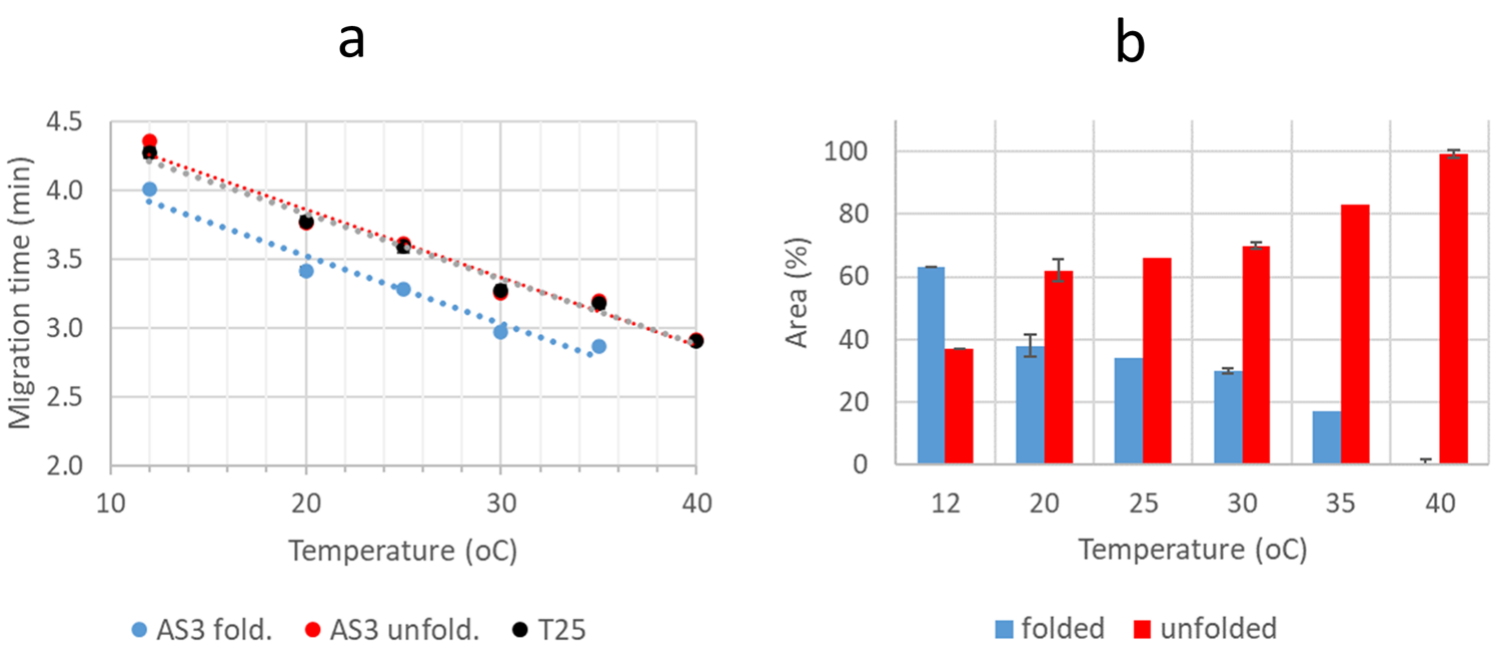
Migration times (*t*_m_) (a) and peak areas (b) of the folded and unfolded TT species from the electropherograms recorded at 254 nm of a 15 μM TT sample with a BGE of 15 mM K_2_HPO_4_ at pH 6.5 and 12, 20, 25, 30, 35, and 40 °C.

The *t*_m_ of the detected species in the different electropherograms are summarized in Table 1, and they were consistent with these peak assignments. More graphically, Figure 3a shows the dependence of *t*_m_ of T25 and TT species at pH 6.5 with temperature. It is clearly observed that the second peak of TT, which appeared at a slightly greater *t*_m_, corresponded to the unfolded species, as these *t*_m_ values fitted almost perfectly with those of the T25 unfolded species. The experimentally measured single wavelength electropherograms for TT samples at pH 6.5 with different temperatures were fitted to the sum of two Gaussian functions to have a rough and rapid estimate of the ratio between the concentrations of the folded and unfolded species (Figure 3b). It can be observed that the temperature at which the ratio was 50% (i.e., melting temperature, *T*_m_) was 18 °C. This value was compared with those determined from spectroscopically monitored melting experiments (Section S4). For TT, the *T*_m_ value obtained by CD and UV were 30.0 ± 0.8 °C and 30.9 ± 0.6 °C, respectively, which agreed quite well with a value determined in a previous work (34 ± 1 °C) in a slightly different medium (150 mM KCl, 20 mM KH_2_PO_4_, pH 6.2).^4^ Anyway, the *T*_m_ value obtained by CE-UV and Gaussian fitting was much lower than that obtained by spectroscopy. Therefore, MCR-ALS was explored to get further insight on the CE-UV experiments at pH 6.5 with different temperatures and eventually a more accurate *T*_m_ estimate.

**Figure 4:**
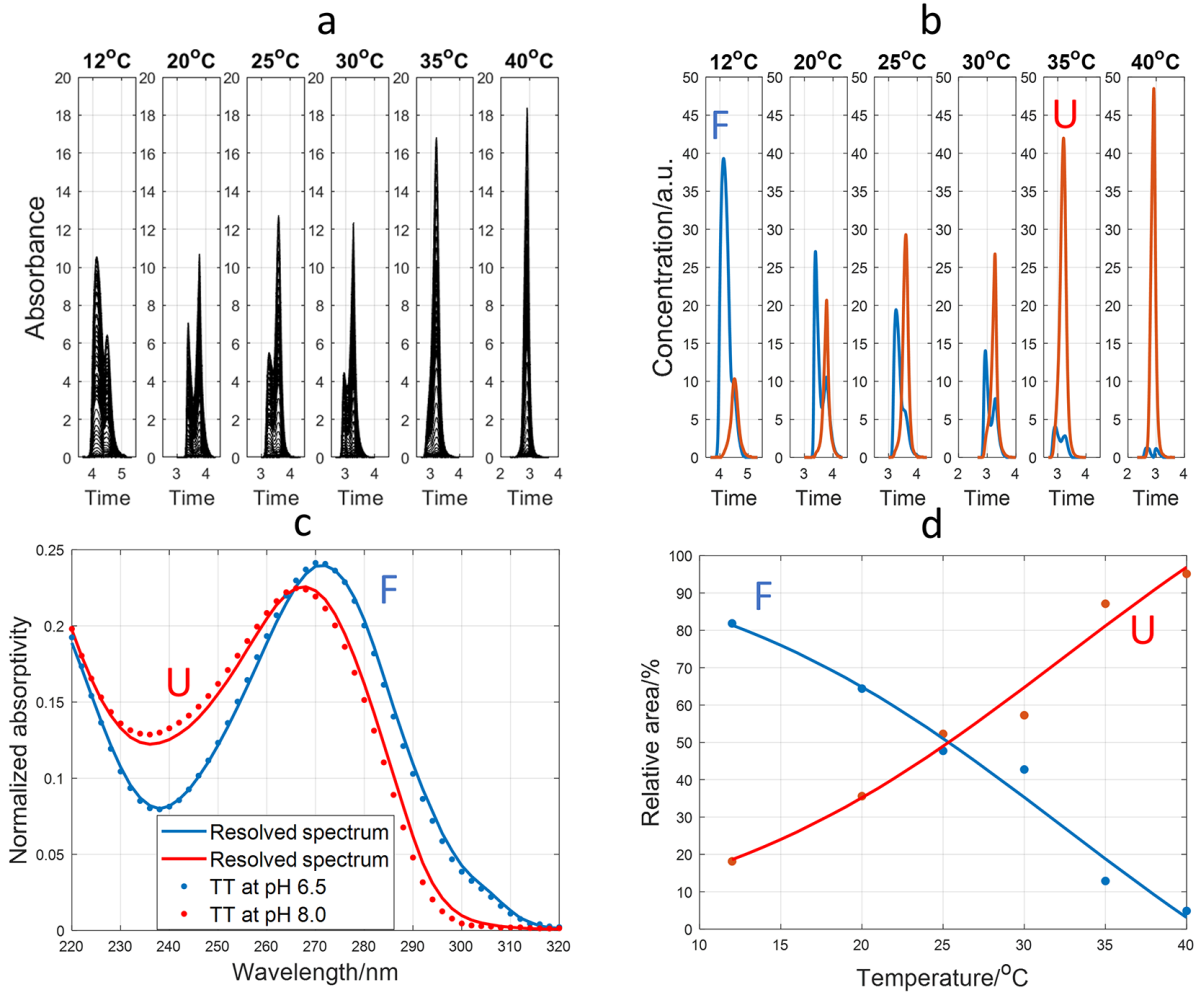
Study of TT folding at pH 6.5 and different temperatures. (a) Measured electropherograms from 220 to 320 nm. (b) Resolved concentration profiles with MCR-ALS with two components. (c) Resolved pure spectra for the two MCR-ALS components. The experimentally measured UV spectra of TT at pH 6.5 and 8.0 (10 °C) were overlapped as symbols. (d) Folded/unfolded species ratio of areas calculated from MCR-ALS concentration profiles. Symbols denote the experimentally measured relative areas, whereas lines are the fitted values using a sigmoidal function. A 15 μM TT sample was analyzed in all cases with a BGE of 15 mM K_2_HPO_4_ at pH 6.5.

The observed small differences in the electrophoretic mobilities and UV spectra levels for folded and unfolded species were considered for the deconvolution and the efficient separation of the i-motif species with MCR-ALS. The goal of this chemometric procedure is the determination of the number of species or components present and the calculation of their corresponding concentration profiles and pure spectra. For TT, the 220–320 nm multiwavelength electropherograms obtained at pH 6.5 and 12, 20, 25, 30, 35, and 40 °C (Figure 4a) were grouped into an augmented data matrix and processed according to Section S2. The analysis of this data matrix was initially done considering the presence of only two components (*Nc* = 2, 0.76% of lack of fit). The calculated concentration profiles and pure spectra of these two components are given in Figure 4b and c, respectively.

The two components considered in the MCR-ALS analysis were assigned to the folded and unfolded TT species (depicted in blue and red, respectively, in Figure 4). This assignment was done by considering the calculated pure spectra as well as the evolution of the concentration profiles with temperature. The pure spectra calculated with MCR-ALS matched those experimentally measured by UV spectroscopy at pH 6.5 and 8.0 and 10 °C for the folded and unfolded TT forms, respectively (Figure 4c). Concerning the calculated concentration profiles (Figure 4b), it was clear that the component depicted in red corresponded to the unfolded species as its concentration increased with temperature. Concomitantly, the component depicted in blue corresponded to the folded species, as its concentration decreased with temperature. From the representation of the ratio of areas of the two MCR-ALS components calculated from the concentration profiles, a *T*_m_ value around 25 °C was obtained (Figure 4d). This *T*_m_ value was closer to those determined spectroscopically, confirming the great performance and reliability of the MCR-ALS deconvolution.

A closer observation of the concentration profile for the folded species in Figure 4b showed the presence of two partially overlapped peaks, which could not be resolved by increasing the number of components in MCR-ALS to 3 because both presented the same spectra. This was rather surprising, as it was expected that the folded and unfolded species would migrate as a single peak. It is known that folding of telomeric sequences, which are very similar to TT, is intramolecular.^41^ To rule out the possibility of multimer formation, CE-UV separations of TT at 2, 5, 15, 25, and 35 μM were carried out. In all cases, the formation of multimeric species was not observed. Therefore, the presence of two peaks for the folded species was assigned to the existence of an additional equilibrium involving the 5′E and 3′E spatial conformers in addition to the equilibrium involving the unfolded species. The TT sequence may form two different conformations because the closing C·C^+^ base pair can either be formed at the 5′-end of the cytosine-rich strand (5′E conformation) or at the 3′-end (3′E conformation). At thermodynamic equilibrium, there is slow conformational refolding from one conformation to the other, which is due to a change in intercalation of the C·C^+^ base pairs (Section S5). In the case of the cytosine-rich sequence corresponding to the human telomer (which is very similar to the TT sequence studied here), the conformational equilibrium has been characterized using a rapid-mixing time-resolved NMR device able to characterize the kinetics of the pH-induced i-motif folding and unfolding from pH 9 to pH 6 and 288 K at atomic resolution.^42^ It has been described that the equilibrium between these two conformers is ruled by rate constants in the order of 10^–3^ min^–1^, whereas folding from the unfolded strand shows rate constants equal to 2 min^–1^ (for the folding into the 3′E conformation), and 0.9 min^–1^ (for the folding into the 5′E conformation). Therefore, the formation of the 3′E conformation from the unfolded strand is faster than that of the 5′E, whereas this last conformation is the predominant once reached equilibrium. Therefore, we concluded that the proposed CE-UV method assisted by MCR-ALS was powerful enough not only to resolve the folding-unfolding equilibrium but also the 3′E to 5′E equilibrium. The equilibrium constants ruling this conformational equilibrium were calculated at several temperatures from the areas of both conformers in Figure 4b, as described in Section S5. These values agreed with the determined value of this constant at 15 °C by NMR.^42^ This is a great advantage over CD and UV spectroscopic techniques, which are not able to monitor such minor structural changes.

### Analysis of Py39WT Sequence

**Figure 5:**
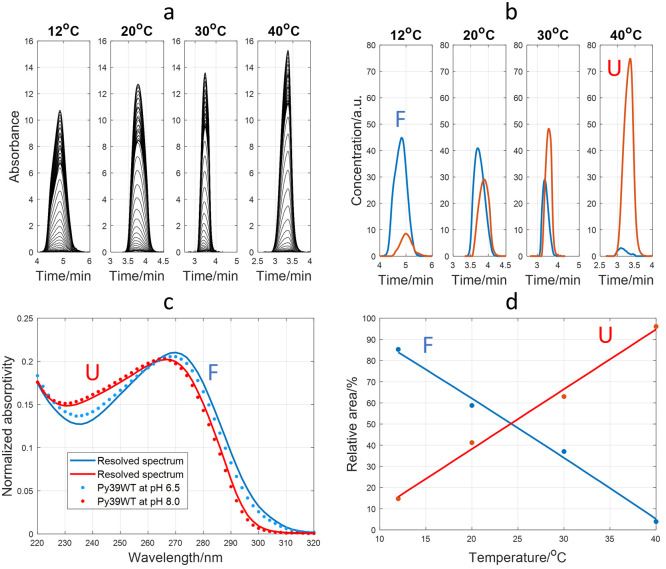
Study of Py39WT folding at pH 6.5 and different temperatures. (a) Measured electropherograms from 220 to 320 nm. (b) Resolved concentration profiles with MCR-ALS with two components. (c) Resolved pure spectra for the two MCR-ALS components. The experimentally measured UV spectra of Py39WT at pH 6.5 and 8.0 (10 °C) were overlapped as symbols. (d) Folded/unfolded species ratio of areas calculated from MCR-ALS concentration profiles. Symbols denote the experimentally measured relative areas, whereas lines are the fitted values using a linear function. A 15 μM Py39WT sample was analyzed in all cases with a BGE of 15 mM K_2_HPO_4_ at pH 6.5.

A similar approach was used to study the folding of the Py39WT i-motif sequence at pH 6.5. In this case, the application of MCR-ALS to the 220–320 nm multiwavelength electropherograms obtained at pH 6.5 and different temperatures was essential because only one peak was observed in the CE-UV electropherograms (Figure 5a). Figure 5b,c shows the resolved concentration profiles and pure spectra considering the presence of 2 components (0.41% of lack of fit), because with 3 components, the third component only modeled a small amount of residual variance (Section S6). The comparison of the resolved pure spectra with those experimentally measured at pH 6.5 and 8.0 (at 10 °C), as well as the *t*_m_ and the evolution of the concentration profiles with temperature, allowed the identification of these components. As in the case of the TT sequence, the component related to the folded species was expected to migrate earlier than the unfolded species, while its concentration decreased with *T*. As the resolved spectrum of this component agreed quite well with that experimentally measured at pH 6.5 and 10 °C, where the i-motif predominates, it was deduced that this component corresponded to the folded i-motif (Figure 5c). Therefore, the second component would be related to the unfolded strand or to a partially folded hairpin.^5,24^ From the plot of the relative areas of the two MCR-ALS components as a function of temperature (Figure 5d), a *T*_m_ value of 24 °C was obtained, which was slightly lower than the values determined by CD or UV spectroscopies (30.2 ± 0.8 °C and 31.0 ± 0.9 °C, respectively, Section S4).

Regarding the presence of a 3′E to 5′E conformational equilibrium, in this case, it was not observed. This may be due to two reasons. First, according to the published i-motif structure formed by Py39WT,^5,24^ there are seven C·C^+^ base pairs. Because of this odd number of base pairs, only one of the conformers (the 3′E) may be formed. Second, the Py39WT sequence is more diverse than the TT sequence, which only showed T and C bases. Because of this, the presence of additional interactions between bases in the case of Py39WT may lock one of the conformers in front of the other.

### Analysis of nmyc01 Sequence

**Figure 6:**
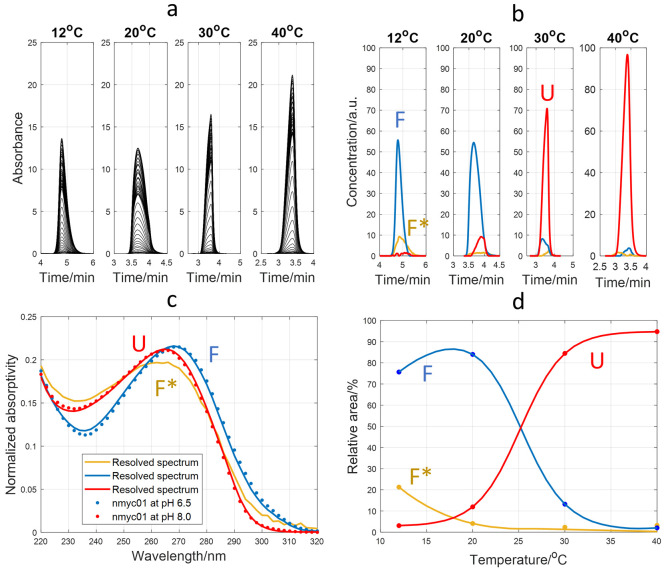
Study of nmyc01 folding at pH 6.5 and different temperatures. (a) Measured electropherograms from 220 to 320 nm. (b) Resolved concentration profiles with MCR-ALS with three components. (c) Resolved pure spectra for the three MCR-ALS components. The experimentally measured UV spectra of nmyc01 at pH 6.5 and 8.0 (10 °C) were overlapped as symbols. (d) Relative areas for the different species calculated from MCR-ALS concentration profiles. Symbols denote the experimentally measured relative areas, whereas lines are the fitted values using sigmoidal functions. A 15 μM nmyc01 sample was analyzed in all cases with a BGE of 15 mM K_2_HPO_4_ at pH 6.5.

Finally, the folding equilibrium of the nmyc01 sequence was also studied. Figure 6 shows the results of the application of MCR-ALS to the 220–320 nm multiwavelength electropherograms obtained at pH 6.5 and different temperatures. In this case, the best results were obtained with three components (0.31% lack of fit), as the third component clearly showed pure spectra and concentration profile compatible with an additional structure (Figure 6b,c). The spectra, *t*_m_, and concentration profiles again allowed for the identification of the species. Two folded species were migrating before the unfolded species, and the resolved spectra for the two most abundant species agreed with those measured experimentally for nmyc01 at 10 °C and pH 6.5 and 8.0, respectively (Figure 6c). Note from Figure 6b that the minor species was only detected at 12 and 20 °C, and it could probably be a relatively stable folded hairpin (Section S5). However, this tentative identification should be experimentally confirmed by other techniques as there is no evidence in the literature. Considering the two most abundant folded and unfolded species, a *T*_m_ of 26 °C was obtained from the plot of the relative areas of the different MCR-ALS components (Figure 6d). CD and UV-monitored melting experiments (Section S4) provided *T*_m_ values of 28 ± 1 and 25.6 ± 0.9 °C, respectively. All together, these values were like that previously determined spectroscopically in a slightly different medium (25 ± 1 °C at pH 6.4, 20 mM phosphate buffer, and 150 mM KCl^6^).

## Conclusions

A novel CE-UV method followed by deconvolution of highly overlapped electrophoretic peaks by MCR-ALS has been described for the evaluation of the folding equilibrium of three cytosine-rich i-motif sequences at different pH values and temperatures. Rapid and repeatable CE-UV analyses have been obtained in reverse polarity of the separation voltage using short capillaries coated with HPC and an appropriate conditioning procedure to avoid DNA adsorption on the inner capillary walls. The CE-UV method has been successfully extended in its capabilities to efficiently separate, detect, and quantify DNA conformers by integrating MCR-ALS. For the TT sequence, the proposed approach has allowed discrimination between the unfolded, and the folded 5′E and 3′E conformations, without the use of expensive techniques like NMR. Similarly, two and three differently folded species have been resolved for Py39WT and nmy01, respectively. In addition, good estimates of *T*_m_ have been obtained for the studied DNA sequences. It has been demonstrated that CE-UV assisted by MCR-ALS enables a straightforward and rapid qualitative and quantitative monitoring of the folding of i-motif structures, including minor species that are missed with routinely applied CD and UV spectroscopic techniques. It provides a highly powerful and inexpensive analytical alternative in the currently available toolbox for investigation of the folding equilibrium of i-motif structures, which may be extended to other DNA structures. In addition, further developments may be made to expand the characterization capabilities of the current approach by improving electrophoretic separation capacity and exploring online mass spectrometry detection.

## Supporting Information Available

Procedures to prepare HPC-coated capillaries and CE-UV analysis, application, and validation of MCR-ALS to analyze i-motif CE-UV data, spectroscopically monitored melting experiments, conformational equilibrium in TT, and MCR-ALS results with three components from Py39WT (PDF)
