# Supplementary material for: Rapid and Highly Efficient Separation of i-Motif DNA Species by CE-UV and Multivariate Curve Resolution
Source: Anal Chem. 2023 Oct 2;95(41):15189–98. doi: 10.1021/acs.analchem.3c01730 (PMC10585953; doi:10.1021/acs.analchem.3c01730)
Supplement: Supplementary file 1 — ac3c01730_si_001.pdf [file ac3c01730_si_001.pdf]

Supplementary Material

Rapid and high-efficient separation of i-motif DNA species by CE-UV and multivariate curve resolution

Laila Bchara[1], Ramon Eritja[2], Raimundo Gargallo[1]*, Fernando Benavente[1,3]*

1. Department of Chemical Engineering and Analytical Chemistry, University of Barcelona, Marti i Franquès 1-11, E-08028 Barcelona, Spain

2. Institute for Advanced Chemistry of Catalonia (IQAC-CSIC), CIBER-BBN, Jordi Girona 18-26, E-08034 Barcelona, Spain

3. Institute for Research on Nutrition and Food Safety (INSA·UB), University of Barcelona, Av. Prat de la Riba 171, E-08921 Santa Coloma de Gramenet, Spain

**Section S1.**	Procedures to prepare HPC coated capillary and CE-UV analysis	p. S2
**Section S2.**	Application of MCR-ALS to analyze i-motif CE-UV data	p. S3
**Section S3.**	Validation of MCR-ALS to analyze i-motif CE-UV data	p. S5
**Section S4.**	Spectroscopically monitored melting experiments	p. S12
**Section S5.**	3'E to 5'E conformational equilibrium in TT	p. S14
**Section S6.**	Resolved concentration profiles and pure spectra with MCR-ALS with three components for Py39WT.	p. S16

Section S1. Procedures to prepare HPC coated capillary and CE-UV analysis

A 5% (m/v) solution of HPC was prepared by heating and sonicating for 1 hour at 40°C. Then, it was kept overnight at room temperature to eliminate bubbles and it was filtered through a 0.22 µm filter before use. A piece of 100 cm long bare fused silica capillary was flushed at 930 mbar using the CE instrument with: methanol (10 min), 1 M KOH (10 min), water (10 min), 1 M HCl (10 min), and HPC solution (1 h). When done, the capillary was cut from both sides about 2 cm to prevent clogging and dried with N_2 gas at 1.5 bar using a set-up consisting of a Kitasato flask connected to a N_2 source and closed with a rubber cup blocked with a clamp. Most of the capillary was outside of the Kitasato flask to be placed inside of a home convection oven (Moulinex OX4448 Optimo, Group SEB, Lyon, France). The oven was conveniently modified to place the capillary inside while leaving the outlet end of the capillary outside in a vial with water to observe N_2 bubbles, which indicated that there was no clogging. After observing bubbles for 10 min, the oven temperature was set and maintained at 140°C for 1 hour while flowing N_2. The temperature was externally monitored with a K-type thermocouple thermometer (Proster TL253, Shenzen, China). Temperature above 160°C must be prevented to avoid polymer degradation. After completing the first layer of coating from the inlet end of the capillary, a second layer was coated from the outlet end of the capillary. To prepare this second layer, the capillary was flushed at 930 mbar from the outlet end using the CE instrument with HPC solution (1 h), and the N_2 flushing and thermal stabilization procedure was repeated to coat from the other end. Once completed the second layer, the capillary segments that were outside of the oven during the heating were cut. Under optimized conditions, 32 cm total length (L_T) capillaries were used for CE-UV experiments. The UV detection window was made at 8.5 cm from the outlet (23.5 cm effective length, L_D). A scalpel was used to remove the polyimide external coating to prevent HPC internal coating damage.

All capillary flushes in CE-UV were performed at 930 mbar. HPC capillaries were conditioned before each analysis with water (10 min), 0.1 M NH_4OH (10 min), water (10 min), and BGE (10 min). Additionally, before the first analysis, new capillaries were equilibrated by applying the separation voltage for 10 min. A separation voltage of 10 kV (reverse polarity, anode in the outlet) was selected to guarantee the Ohm's law fulfillment and an appropriate heat dissipation (the Ohm's law was not fulfilled above 15 kV). All separations were conducted at this separation voltage and temperatures from 12 to 40°C, while keeping the autosampler at the same temperature. This was especially important to avoid great temperature mismatches sample-BGE when working at 12°C. Note that at any of the studied temperatures and with the different BGEs, the electric current was around 50 µA when applying 10 kV, and this could promote a slight increase in the effective temperature inside the capillary (the input power was 1.56 W/m in the 75/375 um id/od capillary), as suggested by Solinova et *al*. (Solinova, V; Kasicka, V, Electrophoresis 2013, 34, 2655–2665). The pressure and time for the hydrodynamic injection was adjusted to inject the same sample volume (~25 nL) at the different temperatures, considering the changes in viscosity and the Hagen–Poiseuille equation [18]. The DNA samples in the different BGEs were injected at 40 mbar for 3 s (12°C), 35 mbar for 3 s (20°C), 30 mbar for 3 s (30°C), and 25 mbar for 3 s (40°C). The electropherograms at 254 nm were monitored during the experiments, while the UV spectra were also recorded scanning from 190 to 400 nm. The BGE of the home vials used for voltage application were refreshed after every analysis. All experiments were repeated at least three times. All samples and solutions were filtered through 0.22 µm filters before use. For overnight storage, the capillary was flushed with water (10 min), 0.1

M NH_4OH (10 min), and water (10 min), to prevent coating damage and avoid salt buildup inside, as well as in the prepunchers and the electrodes. Both ends of the capillary were submerged in vials with water during the storage to avoid drying. For long storage, the capillary was washed in the same way and dried with air.

Section S2. Application of MCR-ALS to analyze i-motif CE-UV data

The most usual way to show CE results is by means of single-wavelength electropherograms (e.g., 254 or 260 nm for DNA). Single-wavelength raw electropherograms were converted to comma-separated value (csv) format with a direct option of the ChemStation software and next imported into Excel 2019 (Microsoft Inc, Redmond, WA, USA) for graphical representation and Gaussian fitting using the Solver complement. In addition, modern commercial CE instruments with UV absorption DAD allow the simultaneous measurement during the separations of absorbance at more than one wavelength (e.g., from 190 and 400 nm as in this study), which produce multiwavelength electropherograms (i.e., multivariate data).

Multivariate data analysis was applied in this study to resolve peaks from i-motif species not completely separated by CE. Multiwavelength CE-UV raw electropherograms were converted to csv format using a macro available with the ChemStation software and next imported into the MATLAB environment (MATLAB R2016a, The Mathworks Inc., Natick, MA, USA). Each electropherogram yielded a matrix of absorbance values with *m* rows (i.e., the *m* times at which absorbance was measured) and *n* columns (i.e., the *n* wavelengths at which absorbance was measured). From the analysis of this matrix, it was possible to determine the number of species or components present in the analysis, as well as their corresponding concentration profiles (i.e., pure migration profiles: absorbance *vs.* time) and pure spectra (absorbance *vs.* wavelength). From the concentration profiles and pure spectra, quantification and identification of the components could be achieved. To reach a good mathematical resolution of these components, their pure spectra should be as much different as possible. For components showing completely overlapped pure spectra, the mathematical resolution may become impossible.

In a mathematical way, it is possible to write:

$\mathbf{D} = \mathbf{C} \cdot \mathbf{S} + \mathbf{E}$ Equation 1

Where **D** is the matrix of the multiwavelength electropherogram (*m* x *n*), **C** is the matrix containing the concentration profiles of each component (*n* x *Nc*, where *Nc* is the number of components), **S** is the matrix containing the pure spectrum of each component (*Nc* x *n*), and **E** is the data not explained by the model (*m* x *n*, which should be close to random noise). This mathematical decomposition may be accomplished by using MCR-ALS.

In this study, MCR-ALS has been applied to the simultaneous analysis of the multiwavelength electropherograms recorded for TT, Py39WT, and nmyc01 at pH 6.5 and different temperatures ranging from 12 to 40°C. According to the requirement for multivariate data analysis, the pure spectra of the *Nc* species (in this case, the folded and unfolded conformations) are invariant in the studied temperature range. Therefore, all spectra measured in these analyses are considered as the result of the linear combination of the pure spectra of the different conformations. This hypothesis

has been shown to be right in the case of analysis of multivariate data recorded along spectroscopically monitored melting experiments.

The scheme of the simultaneous analysis is given in Figure S1. The result of using a column-wise augmented matrix for this analysis will be an augmented matrix containing the concentration profiles of the folded and unfolded species at the four temperatures considered (**C^{12}**, **C^{20}**, **C^{30}**, and **C^{40}**), as well as a matrix **S** that contains their pure spectra. Moreover, another augmented matrix **E** with the data not explained by the model will be obtained. Once the concentration profiles of the species are obtained, their relative concentrations can be estimated from the ratios of the areas calculated for the peaks observed in these concentration profiles. MCR-ALS analysis was carried out following standard procedures for the determination of the number of components (singular value decomposition, SVD) and initial estimates (simple-to-use interactive self-modelling mixture analysis, SIMPLISMA). ALS optimization was performed under non-negativity constraints for concentration and spectral profiles, and spectral normalization (equal length.

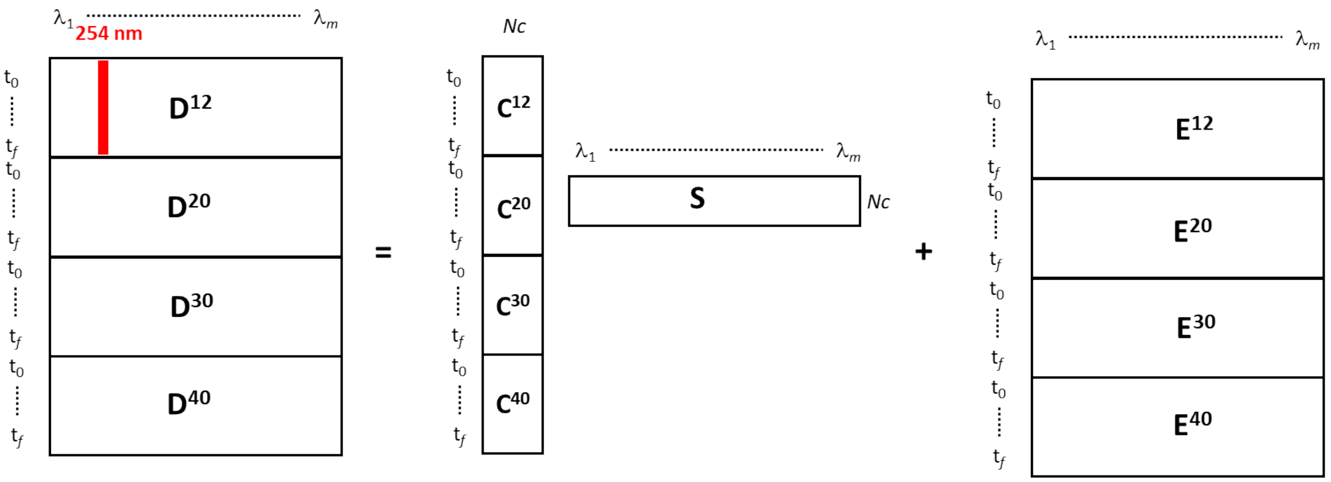

Figure S1. Scheme representing the simultaneous analysis of the multiwavelength electropherograms recorded for the i-motif sequences at pH 6.5 and four different temperatures (as example, 12, 20, 30 and 40ºC). The red rectangle in the matrix **$D^{12\ degrees}$** shows graphically how an electropherogram measured at 254 nm and 12 ºC is located within the column-wise augmented matrix.

Section S3. Validation of MCR-ALS to analyze i-motif CE-UV data

Before analyzing the experimental CE-UV data with MCR-ALS, the performance of this chemometric method was tested. This was done by analyzing an augmented data matrix **D**, which was constructed from a set of simulated concentration profiles of two species ($\mathbf{C_o}$), and their corresponding pure spectra ($\mathbf{S_o}$). If the MCR-ALS analysis of CE-UV data is done correctly, the calculated concentration profiles (**C**) and pure spectra (**C**) with this methodology should match the concentration profiles and pure spectra used previously to construct the data matrix **D**.

This validation procedure is presented here in three main steps.

Step 1. Construction of the augmented data matrix (D) from the simulated concentration profiles ($\mathbf{C_o}$) and pure spectra ($\mathbf{S_o}$).

To construct the augmented data matrix **D** the following steps were followed:

1. The data set was defined (Figure S2). In this case, the matrix **D** contained the simulated data for four experiments, carried out at four temperatures (as example, 12, 20, 30, and 40ºC). In other words, the matrix **D** was a column-wise augmented matrix resulting from merging of four data matrices (each one corresponding to one temperature, named $\mathbf{D^{12}}$, $\mathbf{D^{20}}$, $\mathbf{D^{30}}$, and $\mathbf{D^{40}}$):

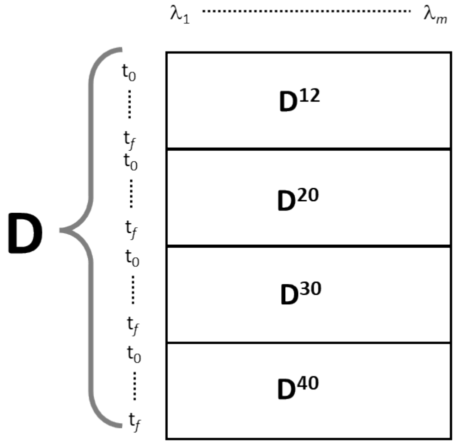

Figure S2. Schematic representation of the data set.

2. The number of species or components (Nc) was defined. In this case, only two components were proposed to be present (Nc = 2) in all experiments. These components corresponded to two hypothetically “folded” (blue, in the following figures) and “unfolded” (red) species. The ratio of the concentrations of these two species was temperature dependent. Hence, at low temperature, the major species was the “folded”, whereas the “unfolded” species predominated at higher temperatures.
3. The simulated concentration profiles were computed. For each experiment, two concentration profiles, corresponding to each one of the two proposed species, were modelled by using the Gaussian function. At 12, 20, 30 and 40ºC, these simulated concentration profiles were named as $\boldsymbol{C_o^{12}}$, $\boldsymbol{C_o^{20}}$, $\boldsymbol{C_o^{30}}$, and $\boldsymbol{C_o^{40}}$ matrices, respectively. Each one of these matrices had dimensions 61 (rows, i.e., times) x 2 (columns, i.e., the number of species). Graphically, these four matrices are shown here (Figure S3):

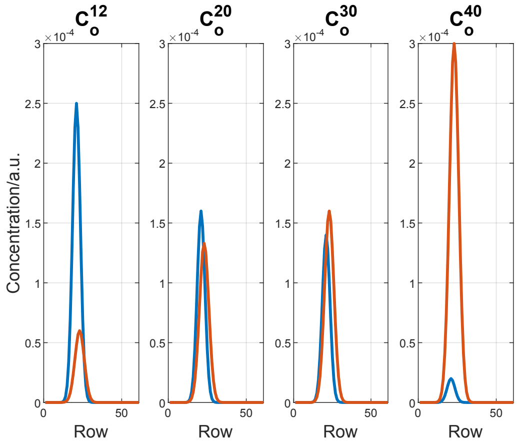

Figure S3. Graphical representation of the simulated concentration profiles.

4. A matrix $\mathbf{S_o}$ containing the pure spectra of the "folded" and "unfolded" species was constructed by using two experimentally measured UV spectra. The dimensions of the $\mathbf{S_o}$ matrix were 2 (rows, i.e., the number of species) x 101 (columns, i.e., 101 wavelengths ranging from 220 to 320 nm with a 1 nm step). Graphically, the matrix $\mathbf{S_o}$ looks like this (Figure S4):

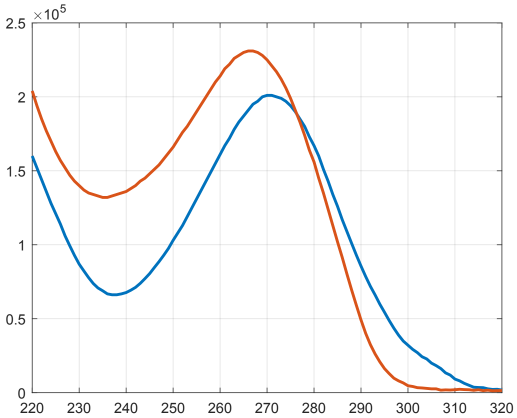

Figure S4. Graphical representation of the pure spectra of the "folded" and "unfolded" species.

5. The matrix $\mathbf{D^{12}}$ was calculated by multiplying the concentration profiles in $\boldsymbol{C_o^{12}}$by the pure spectra in $\mathbf{S_o}$ according to the equation:

$$\boldsymbol{D^{12} = C_o^{12} S_o}$$

Graphically, the resulting data matrix $\mathbf{D^{12}}$ is (Figure S5):

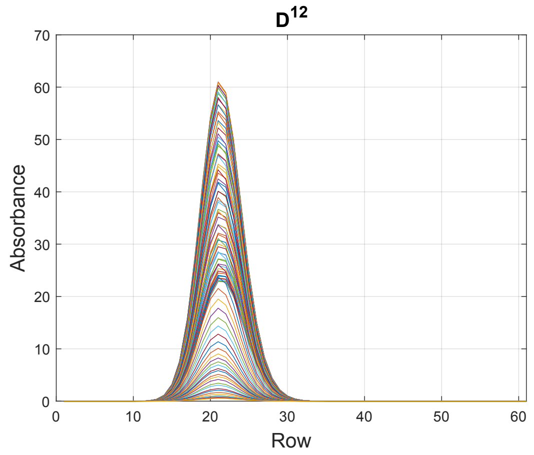

Figure S5. Graphical representation of the data matrix D^{12}.

A similar procedure was used to calculate the other three data matrices $\mathbf{D^{20}}$, $\mathbf{D^{30}}$, and $\mathbf{D^{40}}$.

6. The augmented data matrix **D** was constructed by merging the individual matrices $\mathbf{D^{12}}$, $\mathbf{D^{20}}$, $\mathbf{D^{30}}$, and $\mathbf{D^{40}}$. The dimensions of the matrix **D** were 244 rows x 2 columns. A level of random noise (1%) was also added. Graphically, next figure shows the resulting matrix **D** (Figure S6):

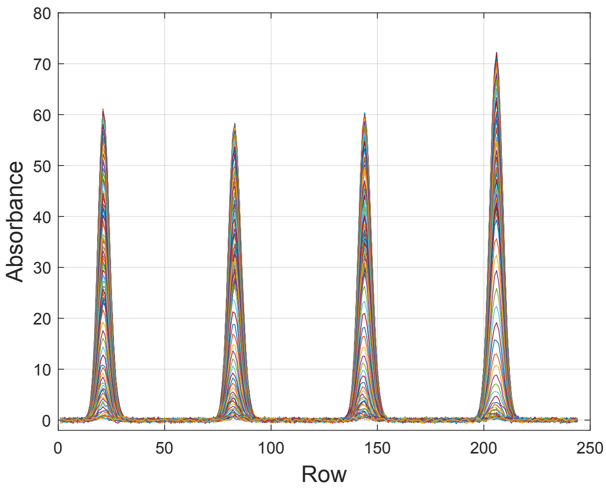

Figure S6. Graphical representation of the augmented data matrix D.

Step 2. Analysis with MCR-ALS of the augmented data matrix D.

When applied to experimental data, the goal of MCR-ALS is the determination of the number of components or species present in the mixture, and the calculation of their concentration profiles and pure spectra. From the calculated concentration profiles, quantification may be possible. Finally, from the calculated pure spectra, qualitative information on the nature of the species may be obtained. For a general matrix, this decomposition is as follows:

$$Data = Concentration\ profiles * \ Pure\ spectra + E$$

Where **E** is data not explained by the model and should be close to random noise.

Graphically, the decomposition of the augmented data matrix **D** is like this (Figure S7):

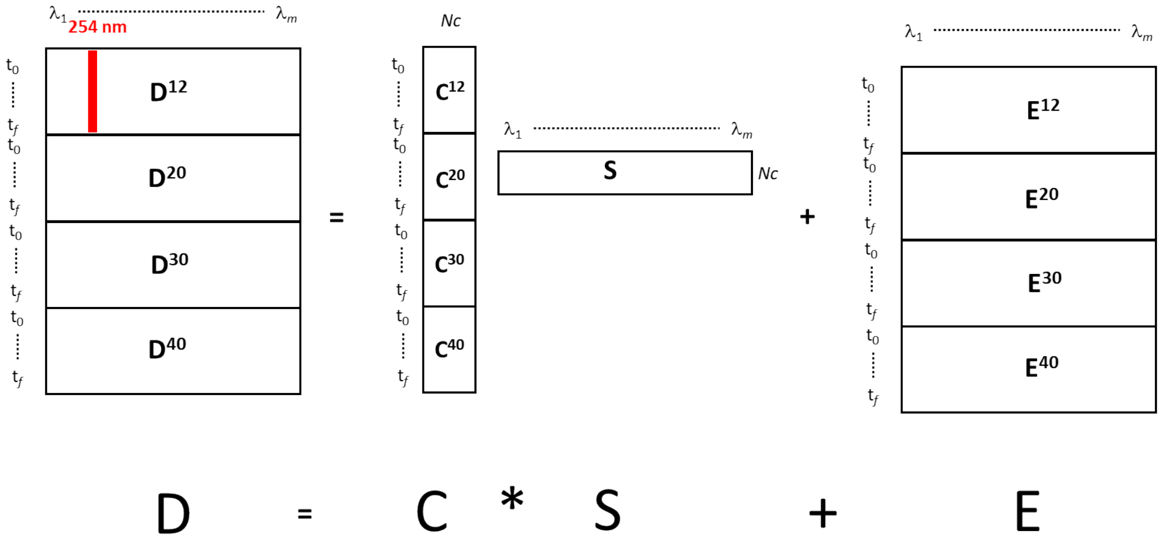

Figure S7. Schematic representation of the decomposition of the augmented data matrix D.

In the case of the validation procedure included here, it is expected that the concentration profiles in matrix **C** and the matrix of pure spectra in matrix **S** calculated with MCR-ALS will be very similar to the concentration profiles and pure spectra in matrices $\mathbf{C_o}$ and $\mathbf{S_o}$, respectively, used in the construction of matrix **D**.

The following actions were followed to decompose the matrix **D**:

1. First, an estimation of the purest spectra was obtained by means of SIMPLISMA method.
2. Second, an iterative process was started where matrices **C** and **S** were calculated in alternate steps. Along this optimization, several constraints were applied, such as the non-negativity of absorptivities in **S** and concentrations in **C**. If no constrains were applied in the iterative process, the number of matrices **C** and **S** that may explain data in matrix **D** would be infinite.
3. The process was finished when a maximum number of iterations is reached or when a convergence criterion is fulfilled.

When analyzing experimental data, MCR-ALS allows the calculation of the analytical (or absolute) concentration of a species present in a mixture ($C_{species\ i}$) from the comparison with the corresponding standard ($C_{standard}$). More precisely, the quantitation is done using this equation:

$$C_{species\ i} = A_{species\ i}\frac{C_{standard}}{A_{standard}}$$

Where $A_{species\ i}$ and $A_{standard}$ refer to the area obtained from to the concentration profiles of the species i and the standard, respectively.

Given the working temperature limitations of the CE instrument used in this work, it was not possible to analyze samples where only the folded or the unfolded species were present, hence standards were not available for an absolute quantification. As an alternative, a relative quantification was done based on the following equation:

$$ratio = \frac{C_{folded}}{C_{unfolded}} = \frac{A_{folded}}{A_{unfolded}}$$

Three different approaches based on this equation were investigated. First, the areas were calculated from the MCR-ALS concentration profiles calculated with the non-negativity constraint of absorbances and concentrations. Second, the pure spectra in **S** were not normalized, normalized to equal height, or to equal length. The resulting concentration profiles and pure spectra calculated in each case with MCR-ALS are given here (Figure S8):

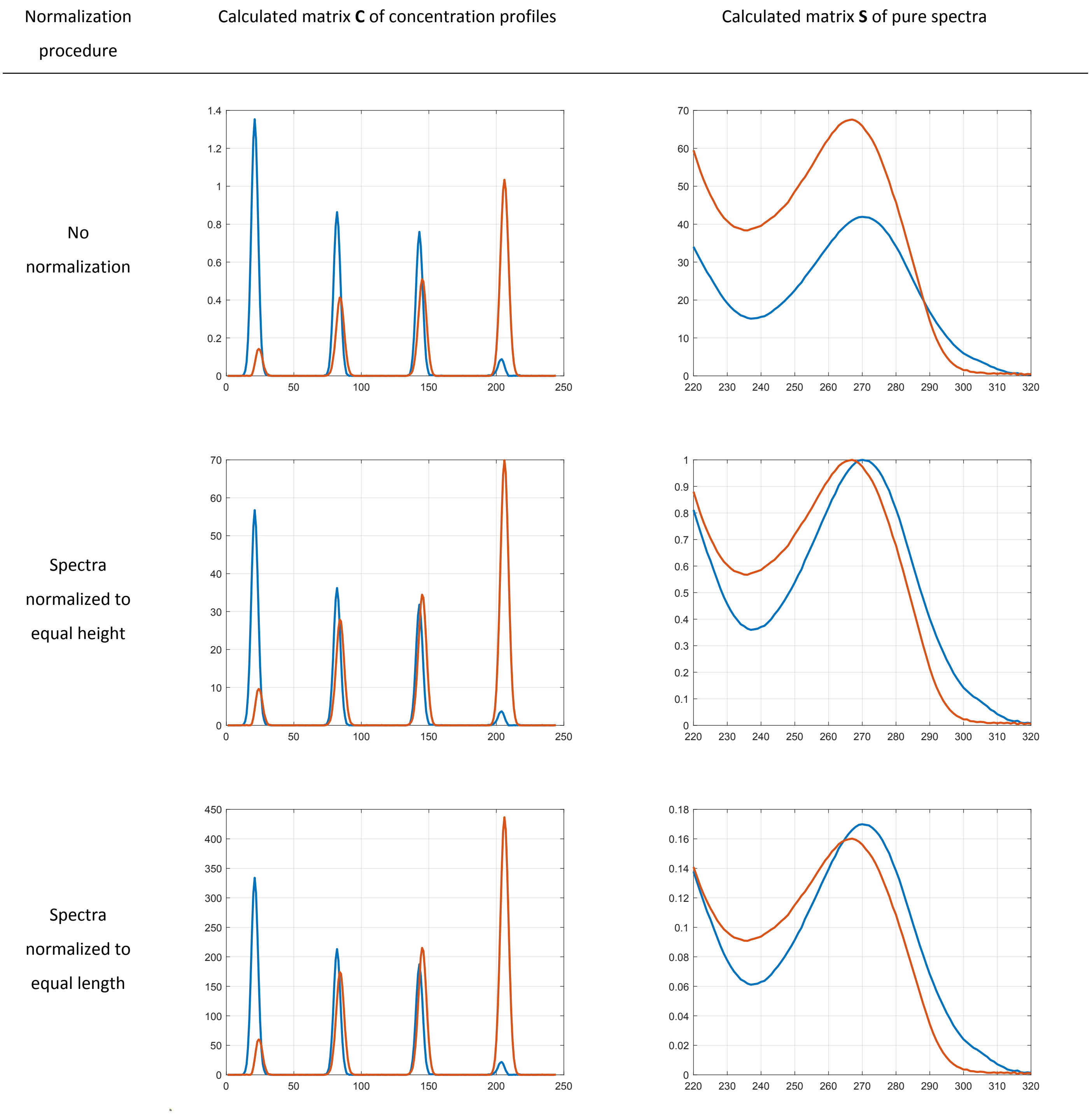

Figure S8. Concentration profiles and pure spectra calculated with MCR-ALS after applying the non-negativity constraint and no normalization, normalization to equal height, and normalization to equal length.

Step 3. Comparison of the simulated and calculated concentration profiles (C_o vs C) and pure spectra (S_o vs S).

Once MCR-ALS was applied to analyze matrix **D**, the calculated concentration profiles (**C**) and pure spectra (**S**) were compared with those used in the simulation (**C_o** and **S_o**). If the agreement is good, the proposed procedure may be considered as validated.

1. Comparison of calculated (**S**) vs simulated (**S_o**) pure spectra.

Apart from visually, it is possible to calculate mathematically the similarity of the calculated pure spectra in **S** matrix with those used in the simulation (**S_o**). For this purpose, the similarity is given by the sine of the angle between two vectors according to the following equation:

$$\sin \alpha = (1 - \cos^2 \alpha)^{1/2}$$

where:

$$\cos \alpha = s_{calculated} \cdot s_{known} / ||s_{calculated}|| \cdot ||s_{known}||$$

The expression "·" denotes the scalar product between both spectra. The expression "$||s_{calculated\ or\ known}||$" denotes the norm of both spectra, computed as the square root of the sum of all squared elements in each spectrum. Using the dissimilarity value ($\sin \alpha$) instead of the similarity value ($\cos \alpha$) provides higher discrimination power for very similar spectra. When both spectra have the same value, $\cos \alpha$ is equal to one and $\sin \alpha$ is equal to zero. A good correlation in shape between two spectra is obtained when the dissimilarity value is lower than 0.0141, which corresponds to a correlation between them greater than 0.99990.

In all three approaches (no normalization, normalization to equal height, and normalization to equal length), the relative intensities of the pure spectra in **S** were different from those of pure spectra in **S_o**. This may be explained because no closure constraint was applied along the iterative process. On the other hand, the shape of the spectrum corresponding to the unfolded species was fully recovered by all three approaches (dissimilarity value was 0.0036). A slightly worse recovery was observed for the spectrum of the folded species (dissimilarity value was 0.0329).

2. Comparison of calculated (**C**) vs simulated (**C_o**) concentration profiles.

In all three approaches, the shape of the original concentration profiles (**C_o**) was recovered. As explained above, the concentration of each species may be calculated from the integration of the corresponding peak. Hence, it was possible to calculate the area ratios for each of the four considered temperatures (12, 20, 30 and 40°C). Table S1 shows the values of the area ratios for C_0 and C, with no normalization, normalization to equal height, and normalization to equal length):

Simulated data

T (oC)	folded	unfolded	folded	unfolded
	absolute	*absolute*	*relative (%)*	*relative (%)*
12	15.63	4.51	78%	22%
20	10.00	10.00	50%	50%
30	8.75	12.03	42%	58%
40	1.25	22.55	5%	95%

Without normalization

T (oC)	folded	unfolded	folded	unfolded
	absolute	*absolute*	*relative (%)*	*relative (%)*
12	8.49	0.91	90%	10%
20	5.37	3.05	64%	36%
30	4.69	3.80	55%	45%
40	0.55	7.75	7%	93%

Normalization to equal height

T (oC)	folded	unfolded	folded	unfolded
	absolute	*absolute*	*relative (%)*	*relative (%)*
12	355.76	61.28	85%	15%
20	225.14	206.41	52%	48%
30	196.82	256.60	43%	57%
40	23.06	524.04	4%	96%

Normalization to equal length

T (oC)	folded	unfolded	folded	unfolded
	absolute	*absolute*	*relative (%)*	*relative (%)*
12	2094.00	382.65	85%	15%
20	1325.20	1289.00	51%	49%
30	1158.50	1602.40	42%	58%
40	135.74	3272.50	4%	96%

Table S1. Values of the area ratios for C_0 (simulatedI and C (calculated) concentration profiles, with no normalization, normalization to equal height, and normalization to equal length).

The best recovery of the areas was obtained when normalization was applied. Slightly better recovery was obtained with normalization to equal length.

Overall, it was concluded that MCR-ALS analysis of CE-UV data provided a good recovery of the concentration profiles and pure spectra used in the simulation. The best results, in terms of recovery of areas, were obtained with normalization to equal length. Good recovery of the pure spectra was achieved in all three approaches. Accordingly, we concluded that the procedure was validated, and the normalization to equal length was applied in the later analysis of experimental data shown in the manuscript.

Section S4. Spectroscopically monitored melting experiments

CD and UV absorption spectroscopies were used to investigate the unfolding of i-motifs formed by TT, Py39WT, and nmyc01 at pH 6.5 (Figure S9). Concerning CD spectroscopy, at pH 6.5 and 5 ^{o}C, all three sequences showed characteristic features related to the i-motif structures, such as the positive band at 225 and 285 nm, and a negative band at 265 nm. Upon heating, the intensity of the bands decreased rapidly, which was related to the unfolding of the i-motif structure. At temperatures higher than 50°C, the CD spectra showed features that could be related to partially folded strands. The melting temperatures (T_m) were determined from the ellipticity curve at 285 nm (see the graph insets).

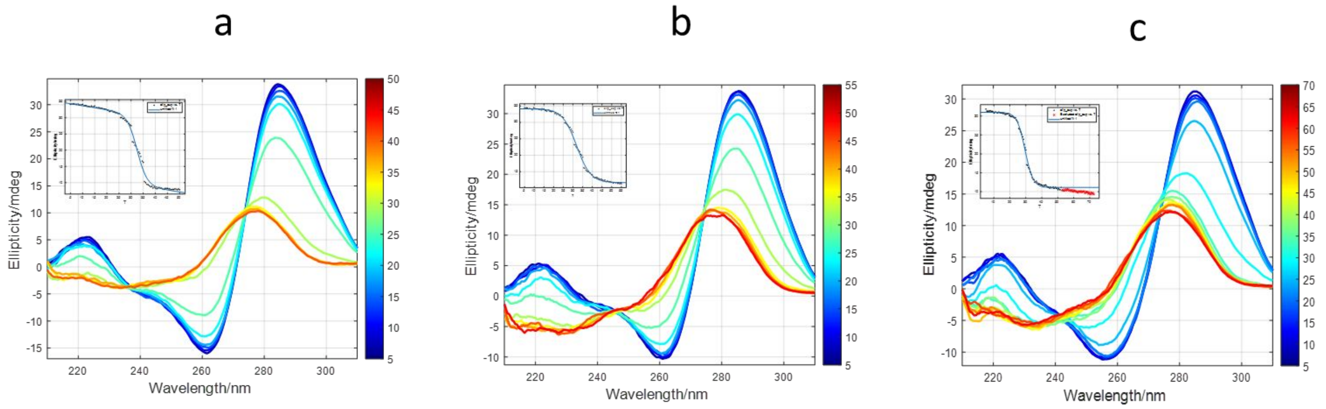

Figure S9. CD spectra measured along the melting experiments at pH 6.5. (a) TT, (b) Py39WT, and (c) nmyc01. Insets show the melting curves measured at 285 nm. A 2 µM DNA sample was analyzed in all cases with a BGE of 15 mM KH_2PO_4 at pH 6.5.

From the ellipticity curve at 285 nm, the melting temperatures T_m were determined. Independent UV-monitored melting experiments were also carried out (Figure S10):

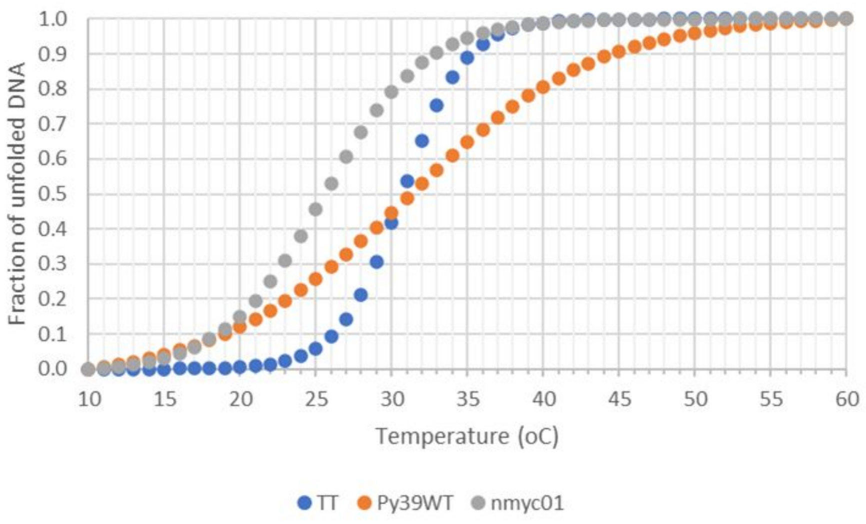

Figure S10. Fraction of unfolded DNA *vs.* T calculated from the absorbance measured at 265 nm at pH 6.5. A 1 µM DNA sample was analyzed in all cases with a BGE of 15 mM KH_2PO_4 at pH 6.5.

In addition to the determination of T_m values, the changes in enthalpy and entropy associated with the unfolding process were also calculated. The determined values agreed with those determined previously (Table S2).

	This work				Previous works			
	**ΔH ($kcal \cdot mol^{-1}$)**	**ΔS ($cal \cdot K^{-1} \cdot mol^{-1}$)**	**T_m (°C)**	**ΔG at 12°C ($kcal \cdot mol^{-1}$)**	**Reference**	**ΔH ($kcal \cdot mol^{-1}$)**	**ΔS ($cal \cdot K^{-1} \cdot mol^{-1}$)**	**T_m (°C)**
TT	-99 ± 10	-328 ± 35	30.5±0.6	-6.0±0.5	4	-91	-295	34 (pH 6.2)
Py39WT	-49 ± 5	-161 ± 18	30.6±0.6	-3.0±0.3				
nmyc01	-83 ± 8	-277 ± 27	26.7±0.9	-4.1±0.4	6	-59	-197	25±1 (pH 6.4)

Table S2. Thermodynamic data for the folding of the i-motif structures calculated from spectroscopically monitored melting experiments. A two-state folding process has been assumed in the calculations. The values are given as average value ± standard deviation (n=2).

Section S5. 3’E to 5’E conformational equilibrium in TT.

Figure S11 shows the scheme of the 3’E (left) to 5’E (right) conformational equilibrium in TT sequence. Other bases than cytosines have not been included in the diagram for the sake of simplicity.

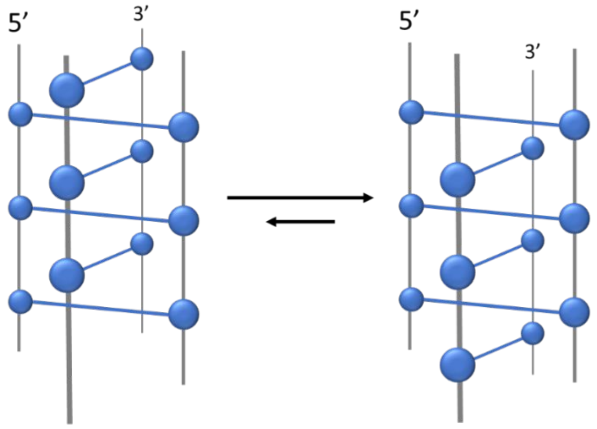

Figure S11. Scheme of the 3’E (left) to 5’E (right) conformational equilibrium in TT sequence

It is possible to calculate the equilibrium constant from the following ratio:

$$K_{eq} = \frac{concentration\ of\ 5'E\ conformer}{concentration\ of\ 3'E\ conformer}$$

If both conformers have very similar molar absorption coefficients, we can write:

$$K_{eq} = \frac{area\ of\ the\ peak\ of\ 5'E\ conformer}{area\ of\ the\ peak\ of\ 3'E\ conformer}$$

The area of each conformer has been measured from the resolved concentration profile for the folded species (Figure 4b in the main text, blue). Table S3 shows the calculated equilibrium constants from the areas of each conformer (the areas are given as relative areas):

Temp. (oC)	1/T	A peak1 (5'E)	A peak2 (3'E)	Keq 5'E/3'E	ln Keq
12	0.00351	68	32	2.13	0.75
20	0.00341	62	38	1.63	0.49
25	0.00335	57	43	1.33	0.28
30	0.00330	54	46	1.17	0.16
35	0.00325	49	51	0.96	-0.04
40	0.00319	59	41	1.44	0.36

Table S3. Calculated equilibrium constants from the areas of each TT sequence conformer (the areas are given as relative areas):

As can be observed, from the values indicated in the article by Lieblein *et al*. (reference 42 in the main text), a K_{eq} for this equilibrium equal to 1.88 was calculated at 288 K (15ºC). This value is between the K_{eq} values found in our work at 12 and 20ºC.

From the slope and intercept of $\ln(K_{eq})$ vs. 1/T, it was possible to determine the changes in enthalpy and entropy that characterize this equilibrium (-6.0 kcal·mol^{-1} and -19.5 cal·K^{-1}·mol^{-1}, respectively) (Figure S12):

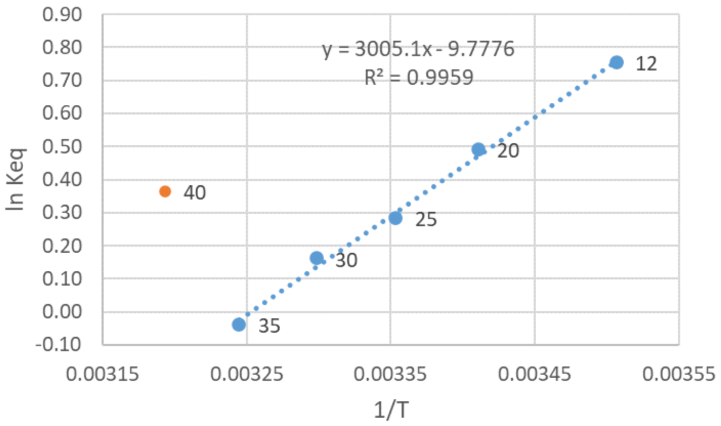

Figure S12. Graphical representation of $\ln(K_{eq})$ vs 1/T for the conformational equilibrium in TT sequence. The labels indicate the temperatures at which the equilibrium constant was calculated.

Note that given the short temperature range, it is expected that the heat capacities remain constant. The calculated value at 40 ºC did not fulfill the model, probably because the areas of the peaks for the 5'E and 3'E have been calculated with high uncertainty at this temperature, as both peaks are very small (Figure 4b in the main text, blue).

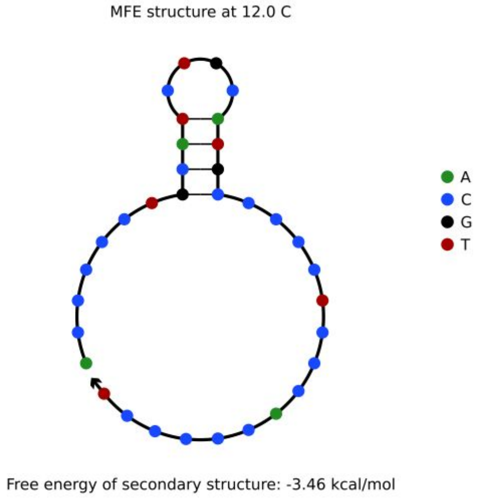

Figure S13. Predicted intramolecular folding of nmyc01 sequence at 12 ºC (J. N. Zadeh, C. D. Steenberg, J. S. Bois, B. R. Wolfe, M. B. Pierce, A. R. Khan, R. M. Dirks, N. A. Pierce. NUPACK: analysis and design of nucleic acid systems. J. Comput. Chem, 2011, 32, 170–173).

Section S6. Resolved concentration profiles and pure spectra with MCR-ALS with three components for Py39WT.

A 15 µM Py39WT sample was analyzed in all cases with a BGE of 15 mM KH_2PO_4 at pH 6.5 and different temperatures.

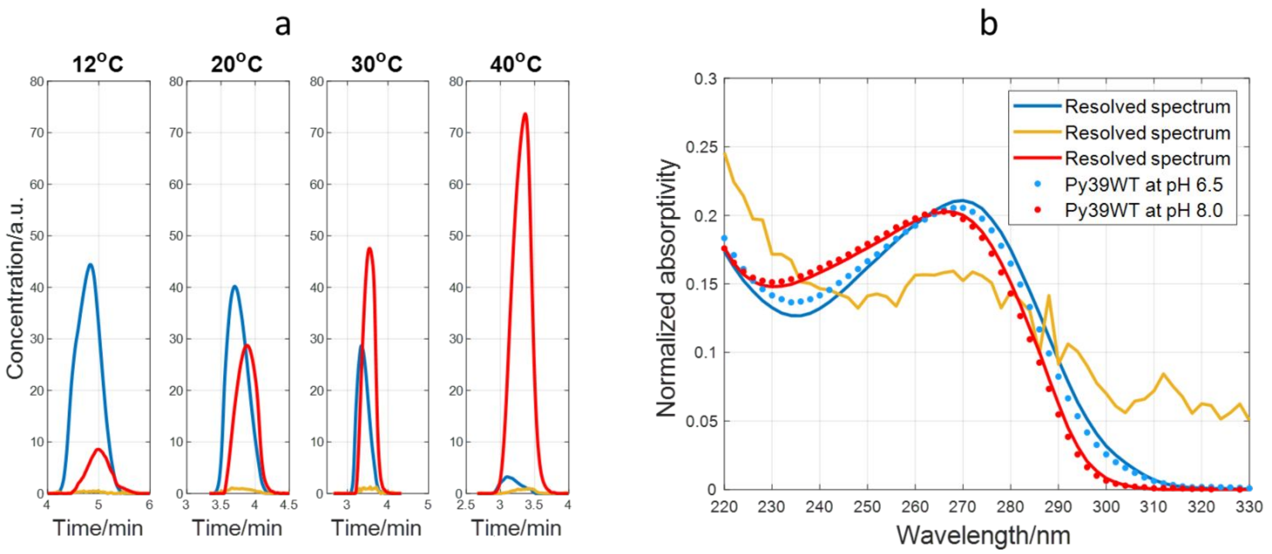

Figure S14. Resolved concentration profiles and pure spectra with MCR-ALS with three components for Py39WT**.**
